# BMPs in periprosthetic tissues around aseptically loosened total hip implants

**DOI:** 10.3109/17453674.2010.492765

**Published:** 2010-07-16

**Authors:** Ville Waris, Eero Waris, Tarvo Sillat, Yrjö T Konttinen

**Affiliations:** ^1^Mikkeli Central Hospital, Mikkeli; ^2^Department of Hand Surgery, Helsinki University Central Hospital, Helsinki; ^3^Department of Medicine, Institute of Clinical Medicine, Helsinki University Central Hospital, Biomedicum, Helsinki; ^4^Department of Anatomy, Institute of Biomedicine, University of Helsinki; ^5^ORTON Orthopaedic Hospital of the ORTON Foundation, Helsinki; ^6^COXA Hospital for Joint Replacement, TampereFinland

## Abstract

**Background and purpose:**

Primary and dynamically maintained periprosthetic bone formation is essential for osseointegration of hip implants to host bone. Bone morphogenetic proteins (BMPs) play a role in osteoinductive bone formation. We hypothesized that there is an increased local synthesis of BMPs in the synovial membrane-like interface around aseptically loosened total hip replacement (THR) implants, as body attempts to generate or maintain implant fixation.

**Patients and methods:**

We compared synovial membrane-like interface tissue from revised total hip replacements (rTHR, n = 9) to osteoarthritic control synovial membrane samples (OA, n = 11. Avidin-biotin-peroxidase complex staining and grading of BMP-2, BMP-4, BMP-6, and BMP-7 was done. Immunofluorescence staining was used to study BMP proteins produced by mesenchymal stromal/stem cells (MSCs) and osteoblasts.

**Results and interpretation:**

All BMPs studied were present in the synovial lining or lining-like layer, fibroblast-like stromal cells, interstitial macrophage-like cells, and endothelial cells. In OA and rTHR samples, BMP-6 positivity in cells, inducible by the proinflammatory cytokines tumor necrosis factor−α and interleukin-1β, predominated over expression of other BMPs. Macrophage-like cells positive for BMP-4, inducible in macrophages by stimulation with particles, were more frequent around loosened implants than in control OA samples, but apparently not enough to prevent loosening. MSCs contained BMP-2, BMP-4, BMP-6, and BMP-7, but this staining diminished during osteogenesis, suggesting that BMPs are produced by progenitor cells in particular, probably for storage in the bone matrix.

## Introduction

Aseptic loosening of total hip replacements (THRs) is the most common reason for revision operations. Connective tissues surrounding aseptically loosened components have been studied since the 1970s to evaluate loosening mechanisms. Peri-implant tissues contain wear debris particles and monocyte/macrophages, foreign body giant cells, and granulomas embedded in connective tissue implant capsule containing collagenous matrix, fibroblasts, blood vessels, and mast cells (Willert and Semlitsch 1977). An extended synovial space and a special synovial membrane-like lining develop upon loosening of implants (Schmalzried et al. 1992). Many of the local resident and immigrant cells have been shown to be activated by using cytokines, growth factors, and other proinflammatory mediators as activation markers and this activation is considered not to be restricted to bone cement due to the so-called particle disease (Santavirta et al. 1992, Harris 1994). Peri-implant foreign body and osteolytic reactions are mediated by fibroblast- and osteoblast-associated receptor activator of nuclear factor kappa B ligand (RANKL, previously known as the osteoclastogenic factor), which activates RANK receptor-positive progenitor cells belonging to the monocyte/macrophage cell lineage to fuse to foreign body giant cells and osteoclasts (Mandelin et al. 2003). This insight has greatly affected implant development so that metal-on-highly cross-linked polyethylene (MoHXPE), metal-on-metal (MoM), ceramic-on-ceramic (CoC), and ceramic-on-metal (CoM) bearings have been developed to reduce volumetric wear and to mitigate particle-driven chronic foreign body inflammation and peri-implant osteolysis.

Less attention has this far been paid to new bone formation, which reflects attempts to achieve, maintain, and/or re-establish osseointegration of the implant in a process that in many respects resembles enchondral bone formation and fracture healing. This point of view seems particularly pertinent when our understanding of osseoconduction and osseoinduction has increased and trabecular metals, hydroxyapatite, teriparatide, and bone morphogenetic proteins (BMPs) have been introduced as tools to stimulate new bone formation. The focus of the present study was on BMPs, because of their essential role in new bone formation and their potential in future therapies.

BMPs were first recognized because of the ability of demineralized and lyophilized bone to induce new bone formation upon implantation in muscle pouches of rabbits. The current name was introduced later, in 1971. BMPs belong to the transforming growth factor-β superfamily of proteins. They have been found to be synthesized in skeletal tissues by osteoblasts and osteoclasts, but also in many extraskeletal tissues by other cells, including fibroblasts, macrophages, and endothelial cells. They act as growth factors, morphogens, or pleiotropic cytokines depending on their spatio-temporal expression and target cells. They can affect cell motility and apoptosis. BMPs drive the differentiation of mesenchymal stromal (stem) cells (MSCs) to bone cells and bone formation. At least BMP-2, BMP-4, BMP-6, and BMP-7 have been shown to be osteoinductive. On top of that, the very same BMPs appear to inhibit osteoclast differentiation and activation (Lories and Luyten 2005). At least some BMPs have also been found to stimulate osteoclasts, a phenomenon that may be linked to bone remodeling. BMP-2 induces osteoclast differentiation and survival (Itoh et al. 2001) and stimulates the formation of osteoclasts in a dose-dependent manner (Wutzl et al. 2006). BMP-4 has stimulatory effects on enzymatic bone resorption of the osteoclasts (Mishina et al. 2004). BMP-5 and BMP-6 have been found to stimulate osteoclast generation in a biphasic mode (Wutzl et al. 2006). BMP-7 induces osteoclastogenesis and recruitment of osteoclasts (Hentunen et al. 1995).

Based on these earlier findings and their ability to promote fracture healing and bone fusion, we studied the above-mentioned osteoinductive BMPs for their eventual presence, cellular localization, and extent of expression in the peri-implant synovial membrane-like interface tissue around aseptically loosened THRs, in control synovial membranes and in MSCs and osteoblasts produced from MSCs.

## Patients and methods

The study protocol was accepted by the Ethics Committee of the Helsinki and Uusimaa Hospital District (5/2000;19.6.2000). Periprosthetic synovial membrane-like interface tissue samples (n = 9) were collected from areas adjacent to osteolytic lesions between uncemented implants or methylmetacrylate cement mantle and host bone from patients undergoing revision THR operation due to aseptic loosening (mean age 76 (68–88) years, 6 women) (Table). No clinical or laboratory signs of infection were detected. Indication for the primary THR operation had been osteoarthritis (OA) in 7 patients, femoral fracture in 1 patient, and rheumatoid arthritis (RA) in 1 patient. Mean time between the primary and revision operations was 10 (4–17) years. Control synovial membrane samples from hip joint capsules were collected from OA patients undergoing primary THR (n = 11, mean age 73 (58–82), 8 women). All samples were fixed in formalin and embedded in paraffin.

**Table:** Data for revision total hip replacement patients

Case	Sex	Age	Diagnosis ^**a**^	Years to revision	Revised component(s)	Method of fixation ^**b**^
1	F	79	OA	13	stem	cemented
2	M	75	Fracture	6	cup, stem	cemented cementless
3	M	88	OA	16	stem	cemented
4	F	68	OA	16	both	cemented
5	M	78	OA	6	both	cemented
6	F	78	OA	5	cup	cemented
7	F	75	OA	7	stem	cementless
8	F	73	OA	4	stem	cementless
9	F	68	RA	17	cup	cemented

^**a**^ OA: osteoarthritis; RA: rheumatoid arthritis.

^**b**^ Method of fixation of revised component(s)

### Immunohistochemistry

5-μm thick paraffin tissue sections were deparaffinized in xylene and rehydrated in a graded ethanol series, and in distilled water. For antigen retrieval, tissue sections were treated with 0.1% pepsin in 10 mM phosphate-buffered 150 mM saline (PBS, pH 7.4) containing 0.01 N HCl for 30 min at 37°C, followed by three 5-min washes with tap water and three 5-min washes with 0.01% Triton X-100 in PBS at room temperature. Non-specific binding sites were blocked with a 1:50 dilution of normal rabbit serum (Vector Laboratories, Burlingame, CA) diluted in 4% bovine serum albumin (BSA) in PBS for 60 min at room temperature. Excess serum was blotted away. For immunostaining, tissue sections were incubated in (1) affinity-purified polyclonal goat IgG to human BMP-2 (sc-6895), BMP-4 (sc-6896), BMP-6 (sc-7406), or BMP-7 (sc-6899) (Santa Cruz Biotechnology, Santa Cruz, CA), all diluted to 2 mg/mL in 4% BSA-PBS, at 4°C overnight; (2) 0.01% Triton X-100 in PBS for 3 periods of 5 min each; (3) biotinylated rabbit anti-goat IgG (Vector Laboratories) for 60 min; (4) 0.01% Triton X-100 in PBS for 3 periods of 5 min each; (5) endogenous peroxidase block solution containing 0.3% H_2_O_2_in methanol for 10 min; (6) 0.01% Triton X-100 in PBS for 3 periods of 5 min each; (7) avidin-biotin-peroxidase complex (Vector Laboratories) for 60 min; (8) Tris-buffered saline (TBS, pH 7.5); and (9) 0.006% H_2_O_2_ substrate and 0.023% 3,3 diaminobenzidine tetrahydrochloride chromogen (Dako ChemMate detection kit; Dako, Glostrup, Denmark) for 150 s. The sections were washed in tap water and one of the 2 sections on each microscope glass slide was counterstained with hematoxylin and the other one was left without counterstain. The sections were dehydrated in graded ethanol series, cleared in xylene, and mounted.

As a negative staining control, normal goat IgG (Jackson Immunoresearch, West Grove, PA) diluted to 2 mg/mL in 4% BSA-PBS was used instead of the primary antibody.

### Photography and microscopic grading

Stained tissue sections were analyzed under 400× magnification using a light microscope coupled to a digital camera. Microscopic findings were graded as follows: 0 = no BMP-immunoreactive cells; 1 = only occasional BMP-immunoreactive cells (< 10%); 2 = moderate numbers of BMP-immunoreactive cells (10–50%); 3 = many BMP-immunoreactive cells (> 50%). Based on their location, arrangement, and morphology, BMP-immunoreactive cells were classified into (1) synovial lining cells, (2) spindle-shaped, stromal fibroblast-like cells, (3) interstitial monocyte/macrophage-like cells, and (4) endothelial cells. Intensity of the staining was evaluated separately. This evaluation was also based in part on our experience in the identification of such cells through our extensive marker studies on interface tissues (Santavirta et al. 1990a, 1990b, 1998).

One tissue sample, approximately 5–10 mm × 5–10 mm, was available per patient. Immunostained and counterstained sections were made sequentially from all tissue sample blocks.

One author (VW) studied each section on 2 occasions with 1 week between, and recorded the above-mentioned BMP-immunoreactivity grades. Sometimes there were slight discrepancies between these 2 recordings, and then a third session was held to define the final BMP-immunoreactivity grade.

### Cell culture

Human passage-4 Poietics MSCs (Lot number 6F4392; Lonza Walkersville Inc., Walkersville, MD) were cultured in Poietics mesenchymal stem cell growth medium according to the instructions of the provider (Lonza). The cells were seeded onto 6-well plates containing coverslips, at 3,500 cells per cm2. For osteogenic differentiation, cells were grown for 14 days in low-glucose DMEM containing 0.1 μM dexamethasone, 50 μM ascorbic acid-2-phosphate, 10 mM β-glycerophosphate (Sigma-Aldrich, St. Louis, MO), and 10% fetal calf serum. Differentiation of the mesenchymal stromal cells to osteoblasts was checked using alkaline phosphatase staining, osteocalcin staining, and staining of the bone mineral as described in detail elsewhere (Myllymaa et al. 2010).

### Immunofluorescence staining

Cells were fixed in 4% paraformaldehyde at room temperature for 20 min, washed twice in 10 mM phosphate buffered 150 mM saline (PBS, pH 7.4) with Triton X100 for 10 min each time, followed by incubations in (1) 5% normal donkey serum (Jackson ImmunoResearch, West Grove, PA) in PBS containing 0.1% bovine serum albumin, for 60 min; (2) 10 μg/mL goat IgG to human BMP-2, BMP-4, BMP-6, or BMP-7 (Santa Cruz Biotechnology) for 60 min; and (3) 10 μg/mL Alexa Fluor 568-labeled donkey anti-goat IgG (Molecular Probes, Eugene, OR) for 60 min. Before mounting, the nuclei were stained with 5 μg/mL 4,6-diamidino-2-phenylindole (DAPI; Vector Laboratories) for 5 min. As negative staining control, non-immune goat IgG (Jackson ImmunoResearch, Newmarket, Suffolk, UK) was used at the same concentration as but instead of the primary antibodies. The cells were observed under a light microscope coupled to a digital camera.

### Statistics

Medians and 95% CIs for medians of cell density scores are presented in Figures 2 and 3. Statistical analysis was done using SPSS software for Windows version 11.5. Mann-Whitney U test was used for comparison of paired variables between revision THR and OA groups because the data were non-parametric. Wilcoxon signed rank test was used for comparison between variables within the revision THR and OA groups using 2-tailed tests.

## Results

### General description of the samples

**Figure 1.**
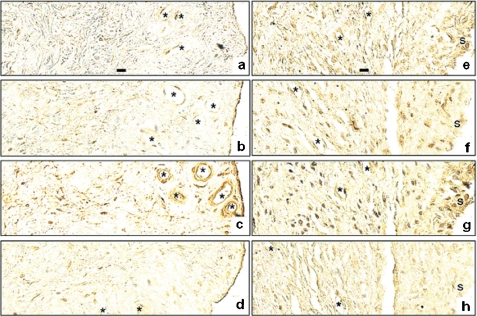
Immunolocalization of BMP-2 (A, E), BMP-4 (B, F), BMP-6 (C, G), and BMP-7 (D, H) in consecutive sections of synovial membrane from the hip joint of one control osteoarthritis patient (A–D) and in consecutive sections of interface tissue surrounding aseptically loosened total hip replacement component from the hip joint of one revision-operated patient (E–H). The synovial lining is present at the right-hand side of osteoarthritis samples (A–D). A similar synovial lining-like layer present in revision total hip replacement samples (E–H) is marked with “s”. Some small arteries and veins are marked with an asterisk. No counterstaining; original magnification ×400. Scale bars (panels A and E only) represent 10 mm.

OA synovial tissue samples contained mostly vascularized connective tissue with spindle-shaped fibroblasts. No foreign bodies or macrophage infiltrates, foreign body giant cells, or granulomas were seen in OA synovial joint capsule samples (Figure 1A–D). Samples collected from revision THR patients consisted of vascularized connective tissue as above, but with infiltrating monocyte/macrophages, foreign body giant cells, and occasionally foreign body granulomas. Particles of metallic debris, polarizing polyethylene particles, and/or polymethylmetacrylate voids were seen in all revision THR samples, both in the extracellular space and intracellularly—phagocytozed by macrophages and foreign body giant cells. A synovial lining or a synovial lining-like layer (marked with “s” in Figure 1E–H) was found to cover the surfaces of the samples.

### Comparison of synovial membrane-like peri-implant tissues in revision THR with control synovial membrane in OA

**Figure 2.**
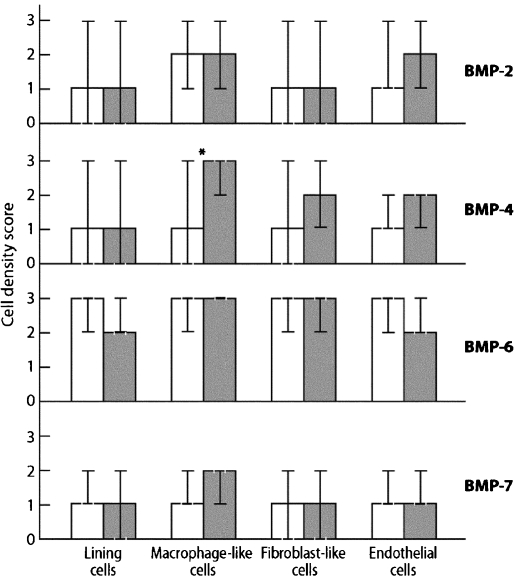
Cell density scores for BMP-2, BMP-4, BMP-6, and BMP-7 in synovial membrane in osteoarthtritis (white bars) compared to synovial membrane-like lining membrane in revision total hip replacement (gray bars). Medians and 95% CIs for medians are presented. The difference in BMP-4 expression between osteoarthritis and aseptic loosening is marked with an asterisk (p = 0.04).

Immunoreactive BMPs were found in synovial linings or lining-like layers, fibroblast-like stromal cells, interstitial monocyte/macrophage-like cells, and endothelial cells in all samples studied. However, the staining patterns were relatively similar in OA control samples and revision THR samples (Figure 2), but there was a difference (p = 0.04) in the density of BMP-4 positive monocyte/macrophage-like cells, which were more frequent in revision THR samples than in OA tissue samples. In addition, the staining intensity of the BMP-4 positive monocyte/macrophage-like stromal cells was stronger in revision THR samples than in OA samples (compare panel B with panel F in Figure 1).

### Comparison of different BMPs

**Figure 3.**
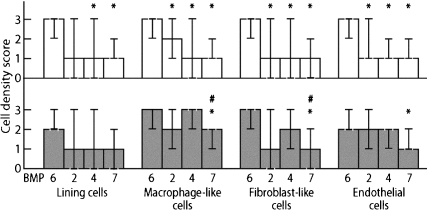
Cell density scores for BMPs (BMP-6, BMP-2, BMP-4, and BMP-7) in synovial membrane in osteoarthritis (upper row, white bars) and in synovial membrane-like lining membrane in revision total hip replacement (lower row, gray bars). Medians and 95% CIs for medians are presented. Statistically significant differences (p < 0.05) between BMP-6 and other BMP types are marked with an asterisk, and statistically significant differences (p < 0.05) between BMP-4 and BMP-7 are marked with #.

When the data were organized according to different BMPs, BMP-6 positive cells were generally more frequent than cells that were positive for other BMPs. BMP-7 positive cells were the least frequent among the BMPs studied, whereas BMP-2 and BMP-4 positive cells were usually present at intermediate frequencies (Figure 3). More specifically, in OA samples BMP-6 positive cells were more frequent than cells positive for BMP-2, BMP-4, and BMP-7 in all cell types (p < 0.05), except in the comparison between BMP-6 and BMP-2 expression in synovial lining cells (p = 0.06). There were no statistically significant differences between the frequencies of BMP-2, BMP-4, and BMP-7 positive cells.

In revision THR samples, BMP-6 positive cells were more frequent than BMP-7 positive cells in fibroblast-like stromal cells (mean 2.6 vs. 0.9, p < 0.05), macrophage-like cells (mean 2.9 vs. 1.6, p < 0.05) and endothelial cells (mean 2.2 vs. 1.3, p < 0.05). Furthermore, BMP-4 positive cells were more frequent than BMP-7 positive cells in fibroblast-like stromal cells (mean 1.9 vs. 0.9, p < 0.05) and macrophage-like cells (mean 2.4 vs. 1.6, p < 0.05). Any other differences between staining results were not statistically significant.

### BMPs in MSCs and osteoblasts

**Figure 4.**
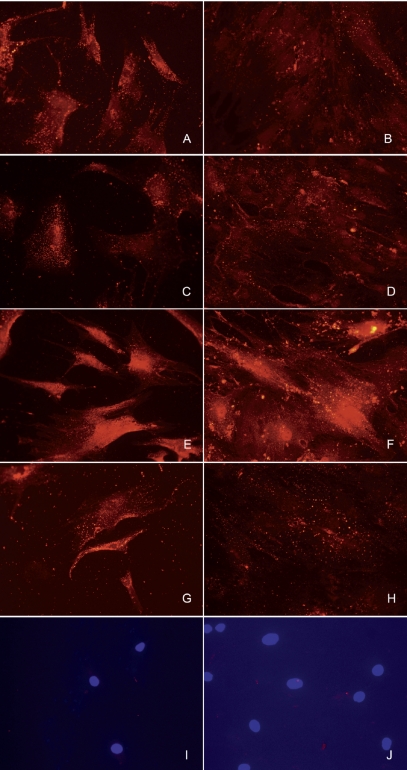
Immunofluorescence staining of undifferentiated bone marrow-derived human mesenchymal stromal/stem cells (A, C, E, G, and I) and after differentiation for 14 days in osteogenic medium (B, D, F, H, and J). Staining for BMP-2 (A, B), BMP-4 (C, D), BMP-6 (E, F), and BMP-7 (G, H). Negative control is staining with normal nonimmune goat IgG (I, J). Nuclear staining with DAPI is shown to demonstrate the presence of cells in the photographed sample fields.

Expression of BMP-2, BMP-4, BMP-6, and BMP-7 was found in human bone marrow-derived MSCs by immunofluorescence staining: as granular, cytoplasmic staining (Figure 4, panels A, C, E, and G). In spite of the fact that during the 14-day-long MSC-to-osteoblast culture, the number of the cells increased through proliferation, osteoblast-rich cell cultures still contained less cytoplasmic BMP-immunoreactive granules (Figure 4, panels B, D, F, and H) than the undifferentiated MSC progenitors (Figure 4, panels A, C, E, and G). Control staining of MSCs (Figure 4I) and of osteoblasts (Figure 4J) using normal, non-immune goat IgG confirmed the specificity of the staining.

## Discussion

Although BMP in human bone and bone cells (Kloen et al. 2003), cartilage (Anderson et al. 2000), and synovial fluid (Honsawek et al. 2009) has been studied, it appears that the published data regarding BMP expression in normal or OA synovial tissues are limited to the studies of Bramlage et al. (2006), who reported on BMP-4 and BMP-5 expression, and of Lories et al. (2003), who reported that there is BMP-2, BMP-4, BMP-6, and BMP-7 (OP-1) expression in synovial membranes. BMP-2 and BMP-6 mRNAs were found to be Ipresent most consistently, and Lories et al. (2003) found them to be especially present in fibroblast-like synovial cells, and also to a lesser extent in monocyte/macrophage-like cells. In this respect, our results confirm and extend these earlier findings. We have not found any earlier reports on the possible presence or cellular localization of BMPs in the synovial membrane-like interface tissue around loosening THR implants. We can now report that BMP-2, BMP-4, BMP-6, and BMP-7 are all present around loosening implants, in synovial lining (probably in fibroblast-like type B and macrophage-like type A lining cells), stromal fibroblast-like cells, and interstitial macrophage-like cells. In addition, we found all these BMPs to be expressed in vascular cells.

Apart from the presence and cellular localization, we also graded the extent of BMP-positive cells in revision THR and compared it to that in OA. Activated macrophages start to produce BMP-4 (Blom et al. 2004). In particular, using human cDNA expression arrays, polyethylene-stimulated human macrophage-like cells have been shown to synthesize BMP-4 (Rader et al. 2002). In accordance with these findings, we found that BMP-4 positive monocyte/macrophage-like cells can be found more frequently in the synovial membrane-like interface membrane around loosened THR implants than in the osteoarthritic synovial membrane. This statistically significant difference is not likely to be only a chance finding associated with multiple statistical comparisons, because there is a clear-cut, particle-mediated pathomechanism to explain the increased number of specifically BMP-4 positive monocyte/macrophages around joint implants compared to OA joints. Thus, this increased number of BMP-4 expressing cells around implants may result from increased monocyte/macrophage cell recruitment (Santavirta et al. 1990a, b) coupled with their local activation by wear debris (Lassus et al. 1998, Ma et al. 2003). There was no significant difference in expression of the other BMPs studied between the study groups, which may indicate that they are not affected by particle-associated disease and loosening of the THR implants. Alternatively, they may be similarly affected in both OA and in loosening because their involvement has been implicated in the development of OA (Zoricic et al. 2003, Blom et al. 2004, Badlani et al. 2008)

Interestingly, Lories et al. (2003) reported that expression of BMP-2 and BMP-6 is upregulated in synovial fibroblast-like cells by the proinflammatory cytokines tumor necrosis factor-α and interleukin-1β, which are mostly produced locally by activated macrophages. BMP-2 and BMP-6 were not upregulated by interferon-γ, which is mostly produced by activated T lymphoblasts. In contrast, proinflammatory lipopolysaccharide was found to lead to a reduction in BMP-2 production in human macrophages (Champagne et al. 2002). These differential effects of wear debris particles and soluble proinflammatory factors on fibroblast- and macrophage-like cells may explain why the cytokine-inducible BMP-6 stained more intensively (compared to other BMPs) in so many cells in both study groups. It has also been reported that BMP-7 is only found in lymphoid cells (Detmer et al. 1999), which might explain why BMP-7 positive cells were found so infrequently.

BMPs are involved in osteoinduction and fracture healing because of the ability of presynthesized BMPs to be deposited in the bone matrix and to be released from there during osteolysis (Kloen et al. 2003). Such bone matrix deposition suggests that preosteoblastic MSCs and/or osteoblasts themselves produce BMPs for these stromal deposits. Furthermore, MSCs may also play a role in implant fixation and loosening as potential osteoblast progenitor cells. We therefore checked whether MSCs and osteoblasts produced by osteogenic differentiation from MSCs contain BMPs. Both MSCs and osteoblasts contained BMPs, but MSCs more than osteoblasts, suggesting that MSCs in particular are a potent source of BMPs. This confirms and extends earlier findings. During fracture healing, osteoblasts have been shown to contain BMP-2, BMP-3, BMP-4, and BMP-7, and also their receptors (BMPR-IA, BMPR-IB, and BMPR-II) as well as signs of autocrine and paracrine stimulation in the form of pBMP-R-Smads (Kloen et al. 2003). During the preparation of this work, Seib et al. (2009) published that MSCs produce BMPs, of which BMP-2, BMP-4, and BMP-6 were tested for, and that this may explain why osteogenic differentiation of MSCs forms their default pathway in long-term in vitro culture. Taken together, these findings suggest that MSCs and osteoblasts also constitute a potential source of BMPs in peri-implant bone.

There are 2 simple and straightforward but contradictory interpretations for the difference in BMP-4 between revision THR and control samples. It may be that the particle-induced BMP-4 production, although clearly increased, is not able to promote osseous differentiation enough to maintain implant osseointegration. However, BMPs, including BMP-4, have also been implicated as stimulants in osteoclast formation, activation, and/or function (Mishina et al. 2004). It could be that the relatively high degree of BMP-4 expression in revision THR interface samples is in part responsible for the enhanced activity of osteoclasts, and in fact stimulates peri-implant osteolysis rather than osseointegration. Furthermore, BMPs have been implicated in many diverse responses and their activity is affected by their concentration, other growth factors/cytokines, and mechanical stimuli (for some examples, see Cunningham 1992, Zeisberg et al. 2003, Csiszar et al. 2008, Meynard et al. 2009), so our findings might also be explained by connective tissue growth and by BMP-driven formation of interface tissues. It remains to be seen whether the promise of BMPs in bone defect filling (Murakami et al. 2003), in-growth into porous coating (Bragdon et al. 2003), and fracture healing (Govender et al. 2002, Jones et al. 2006) can be used in future to improve osseointegration of prosthetic joints.

Some limitations of our study are the small sample size and the fact that the histopathological grading was done by only one researcher. Probably the best control material for revision THR samples would be hip pseudocapsule samples surrounding well-fixed total hip replacement implants, but for obvious reasons such samples are difficult to get. Synovial joint samples from osteoarthritic joints or traumatized joints have been used in almost all previous studies as control samples. Finally, the inter- and intra-sample variation in the histopathology of interface tissues is quite high. We therefore used a relatively crude ad hoc grading system, which may have reduced the sensitivity of the analysis, but it was less likely to lead to false results and interpretations due to the topographical variation in tissue histology.
